# Multiple Primary Malignant Neoplasms: An Unusual Case of Metachronous Breast Ductal and Squamous Cell Carcinomas

**DOI:** 10.7759/cureus.6954

**Published:** 2020-02-11

**Authors:** Mohd Elmugtaba Ibrahim, Mohammed Saleh, Ahmed Ali, Navid Alavi

**Affiliations:** 1 Internal Medicine, Howard University Hospital, Washington, D.C., USA; 2 Oncology, Howard University Hospital, Washington, D.C., USA

**Keywords:** multiple primary malignant neoplasms, breast cancer, squamous cell cancer

## Abstract

Multiple primary malignant neoplasms (MPMN) are generally defined as the co-occurrence of multiple primary malignant neoplasms of distinct histology in the same individual. Second and higher-order primary malignancies now comprise about 18% of all incidence of cancer in the USA. The incidence ratio of developing multiple primary cancers (MPCs) in female cancer survivors is 1.17 to 1.6. In women with breast cancer, the incidence ratio is even higher, according to age at diagnosis of breast cancer. However, the concurrence of breast cancer and squamous cell carcinoma is not described in the literature. Primary squamous cell carcinoma of bone is also rare in the skeletal system other than in the skull, with only three such cases reported in the English literature.

We present a case of a 59-year-old woman with high-grade primary invasive ductal carcinoma of the breast and second distinct squamous cell carcinoma metastasis to the bone of unknown primary site. A search for a primary squamous cell carcinoma, including CT head and neck, CT chest, colposcopy, esophagogastroduodenoscopy (EGD) and colonoscopy, did not show any evidence of a primary site.

## Introduction

Multiple primary malignant neoplasms (MPMNs) are a well-recognized entity with an increasing incidence and now comprise about 18% of all incidence of cancer in the USA, with incidence higher in female cancer survivors, in particular those with breast malignancies.

Females being treated for breast cancer have an increased risk of developing leukemia, ovarian cancer, and gynecological cancers, and a slightly enhanced risk of gastrointestinal (GI) cancers, in addition to the well-known risk of developing sarcomas and lung cancer after radiation therapy - there is increased incidence of leukemia in patients receiving chemotherapy (alkylating agents) and gynecological cancers in those receiving hormone therapy (mainly tamoxifen). Another frequent malignancy in breast cancer patients is thyroid cancer.

However, the concurrence of breast cancer and squamous cell carcinoma has never been described in the literature.

## Case presentation

We present a case of a 59-year-old woman with a mammographic finding of a right breast mass. An ultrasound-guided core needle biopsy revealed a diagnosis of moderately differentiated invasive ductal carcinoma, which was ER-positive, PR-positive and HER2/neu-negative. A subsequent excision lumpectomy of the right breast mass revealed a high-grade invasive ductal carcinoma. Invasive ductal carcinoma metastasis was seen in one axillary lymph node, the pathological staging was pT2N1a. The patient received postoperative chemotherapy, four cycles of Adriamycin and cyclophosphamide followed by Taxotere. She did not receive adjuvant radiation therapy or hormonal therapy.

**Figure 1:**
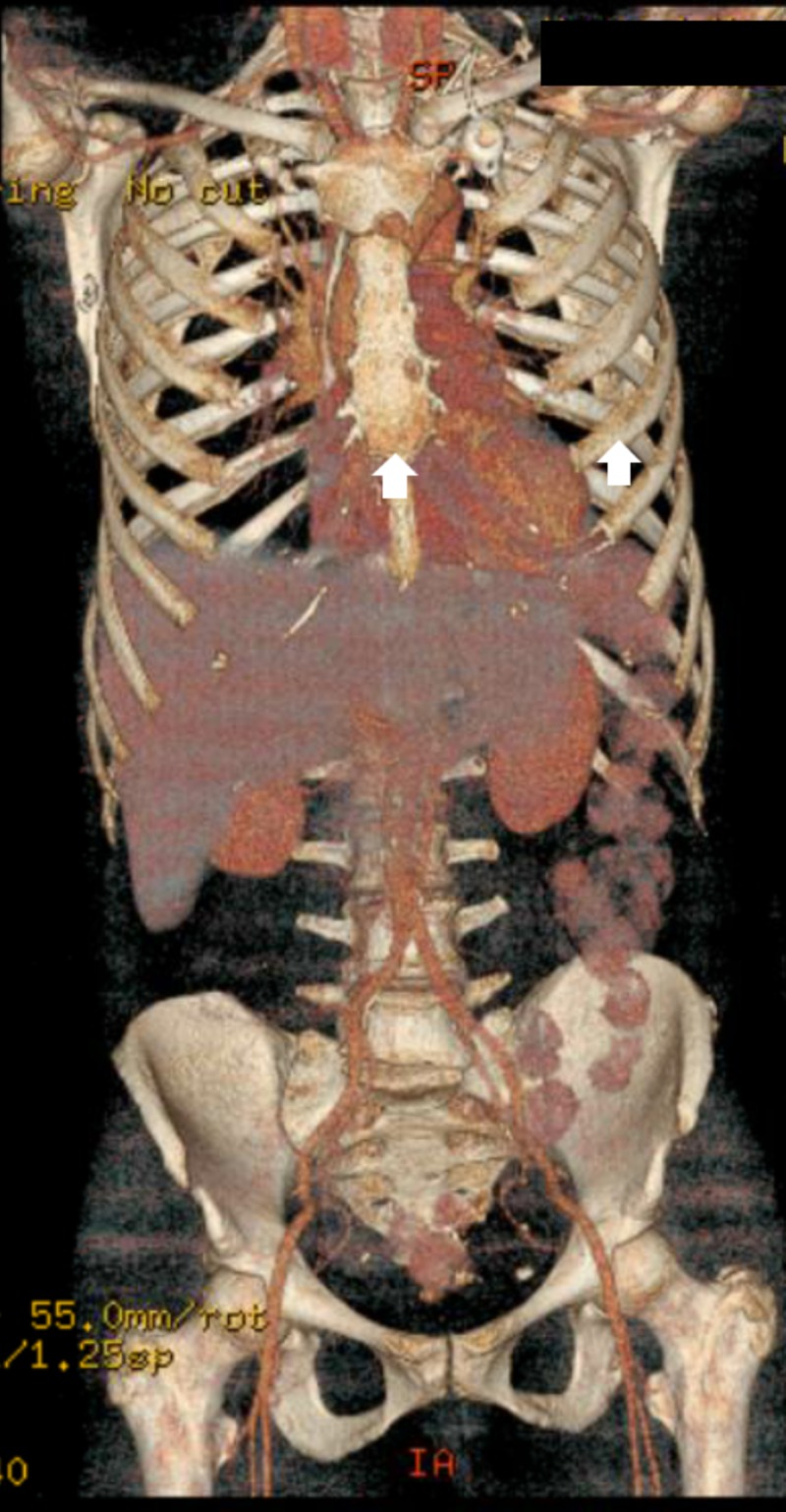
PET-CT scan for staging A PET-CT scan for staging showed diffuse bone involvement in the sternum, ribs (depicted with white arrows) and pelvic bones, no visceral disease.

**Figure 2:**
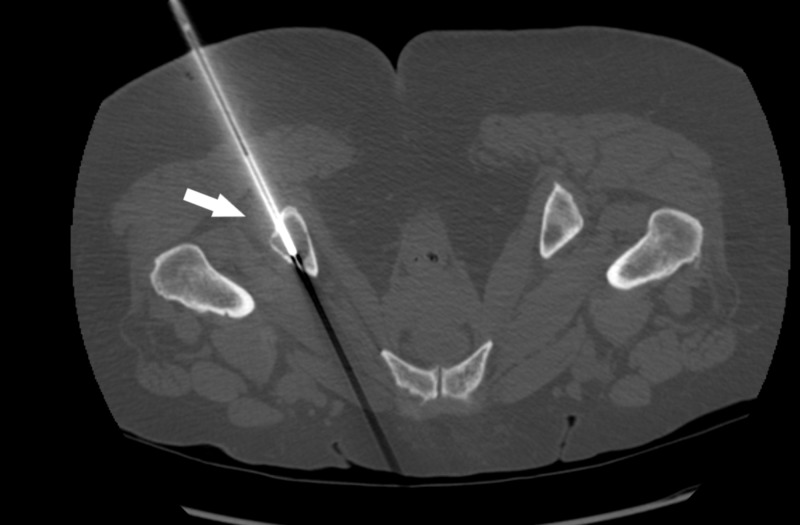
CT-guided biopsy CT-guided percutaneous biopsy of a lytic lesion in the right ischial tuberosity (white arrow).

**Figure 3:**
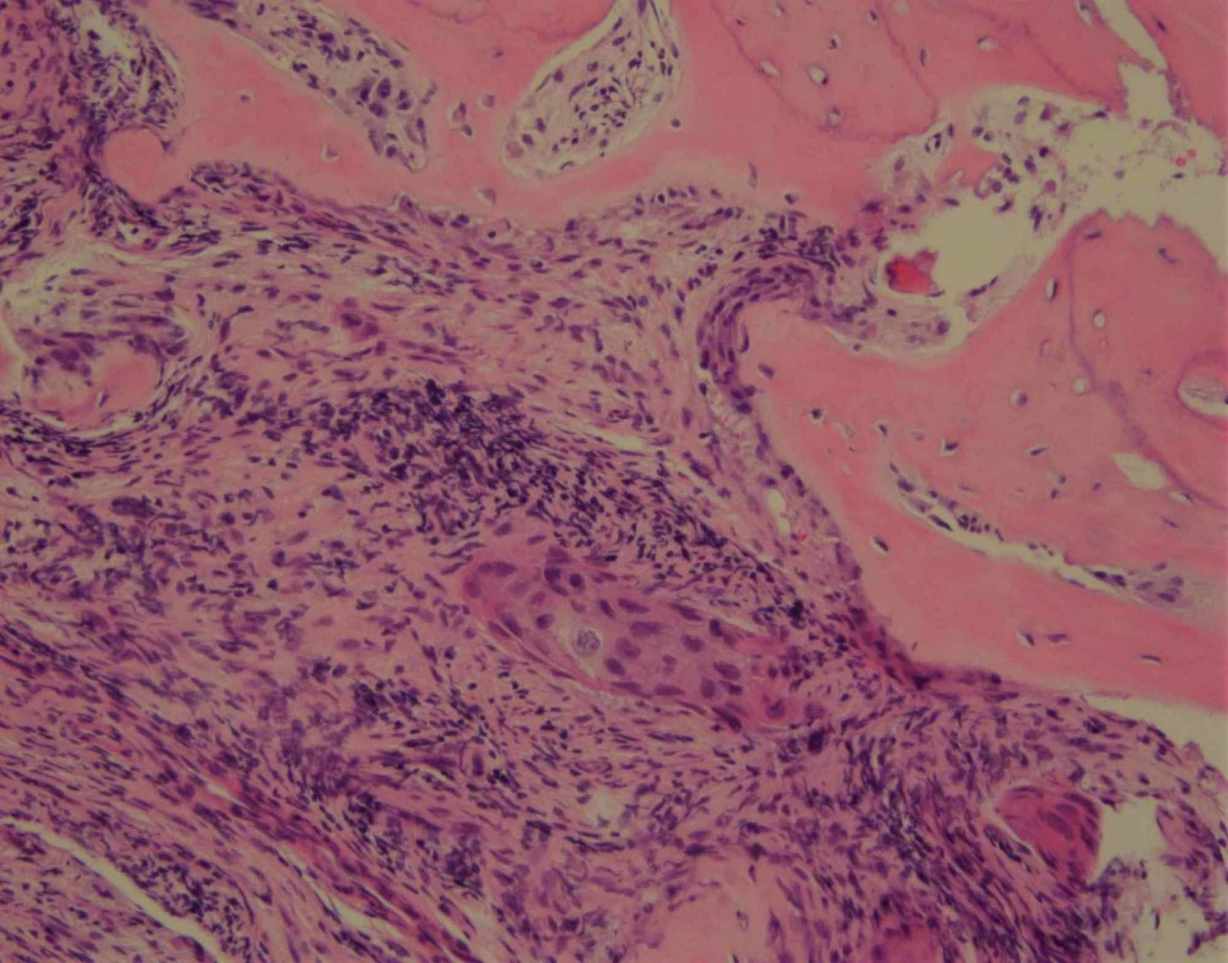
Biopsy fragment from Ischium bone reveals metastatic squamous cell carcinoma

**Figure 4:**
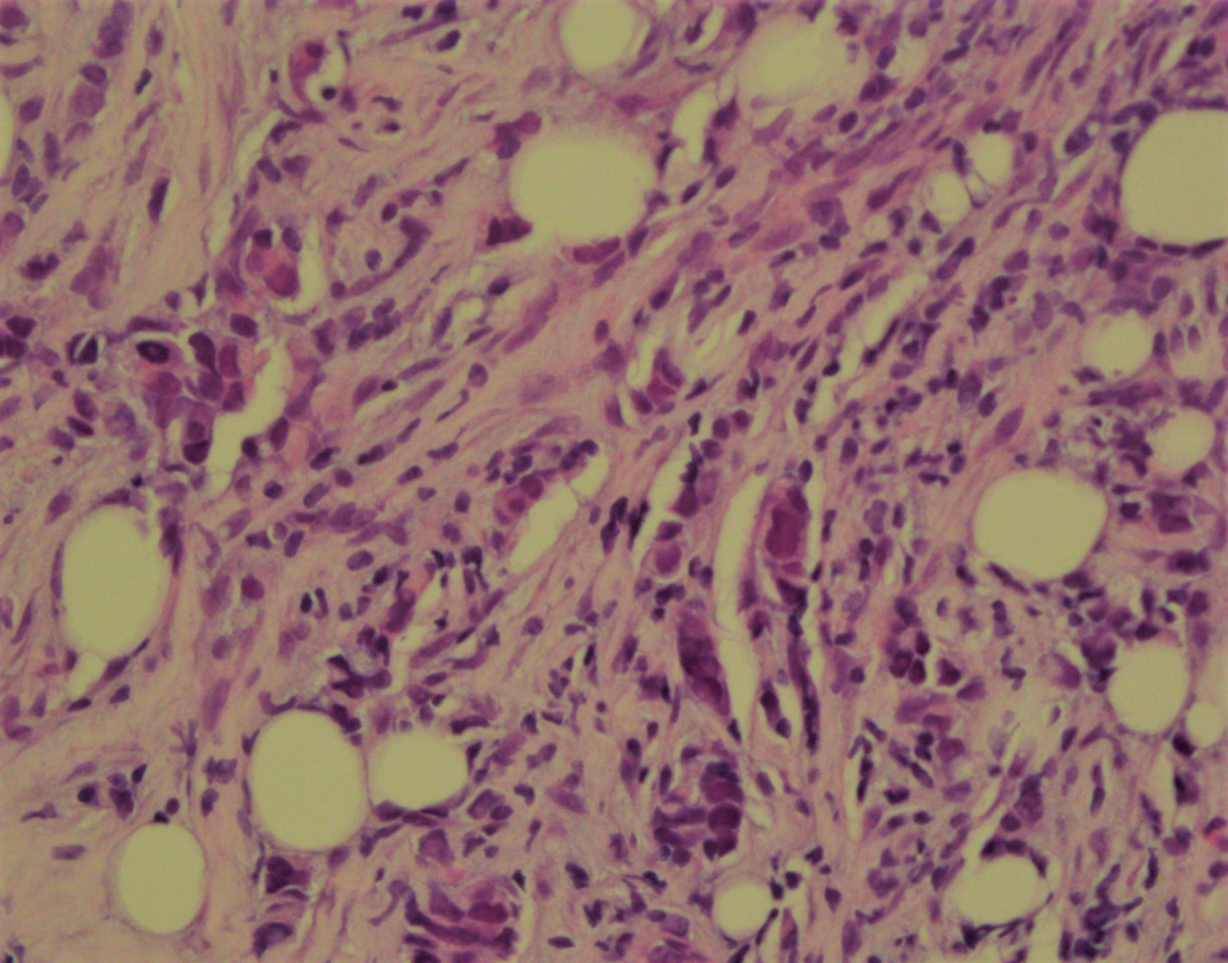
Invasive poorly differentiated ductal carcinoma. Nottingham Grade 3 of 3

She later presented with the recurrence of the entire right breast. A PET-CT scan for staging showed diffuse bone involvement in the vertebral column, ribs and pelvic bones, no visceral disease (Figure 1). She underwent CT-guided percutaneous biopsy of a lytic lesion in the right ischial tuberosity (Figure 2). The pathology revealed metastatic squamous cell carcinoma which was negative for breast origin markers, namely mammaglobin and SOX-1, which favored a diagnosis of squamous cell carcinoma of unknown primary site (Figure 3). A metastatic workup for unknown primary site including CT head and neck, colposcopy, esophagogastroduodenoscopy (EGD) and colonoscopy were all negative (Figure 4).

## Discussion

MPMN was first described in the 19th century by Billroth [1]. It is a well-recognized entity with an increasing incidence ever since. MPMN is generally defined as the co-occurrence of multiple primary malignant neoplasms in the same individual. These tumors must be histologically distinct, exhibit definite malignant features and the possibility that the one is a metastasis of another must be ruled out in order to meet the diagnostic criteria. MPMNs can be either synchronous, when two or more malignancies occur simultaneously over the span of a six-month period, or metachronous, when the interval exceeds the six-month period.

In general, a diagnosis of cancer confers a 20% higher risk of developing a new malignancy. It is estimated that one-third of cancer survivors above the age of 60 were diagnosed with another malignancy [2]. The annual incidence of multiple primary malignancies is variable and is estimated to be anywhere from 0.73 to 11% depending on the study population [2].

Second and higher-order malignancies now comprise about 18% of all cancer incidence in the USA [3]. The incidence ratio of developing MPMNs in female cancer survivors is 1.17 to 1.6 [4]. In women with breast cancer, the incidence ratio is even higher (1.96) (95% confidence interval (CI), 1.48-2.44) according to age at diagnosis of breast cancer [4].

The increased risk of MPMNs in cancer survivors compared to the general population appears to be due to complex factors including genetic predisposition, host factors, environmental determinants, gene-environment interactions, shared lifestyle factors (e.g., tobacco use or excessive alcohol intake), and the late effects of cancer treatments (e.g., cytotoxic, radiation, or hormonal therapies) [3].

Females being treated for breast cancer have an increased risk of developing leukemia, ovarian cancer, and gynecological cancer, and a slightly enhanced risk of gastrointestinal (GI) cancer, in addition to the well-known risk of developing sarcomas and lung cancer after radiation therapy - there is an increased incidence of leukemia in patients receiving chemotherapy (alkylating agents) and gynecological cancers in those receiving hormone therapy (mainly tamoxifen). Radiation therapy alone also has a significant, but lesser, effect found only in comparison with the general population [5-9].

Another frequent malignancy in breast cancer patients is thyroid cancer. A large retrospective study in Korea has shown that thyroid cancer was the most prevalent malignancy among Korean breast cancer patients [4]. However, the concurrence of breast cancer and squamous cell carcinoma is not described in the literature.

Another unusual finding is the site of the squamous cell carcinoma. The most common squamous cell primaries with bony metastasis are those that originate in the lungs, head and neck. Bone metastasis of squamous cell carcinoma is seen in 30-40% of those with advanced lung cancer and only seen in 10% of all head and neck squamous cell carcinoma [10,11]. Primary squamous cell carcinoma of bone is rare in the skeletal system other than in the skull, with only three such cases reported in the English literature [12-14].

## Conclusions

In conclusion, we present a rather unusual case of metachronous MPMN consisting of a primary breast invasive ductal carcinoma and a squamous cell carcinoma of unknown primary site. MPMNs present a complex and frequent problem to cancer patients, and particularly to breast cancer patients. A thorough understanding of the risk factors for developing MPMNs, risk stratification and prevention, is related with the increased life expectancy of many cancer patients with advanced therapeutics.
